# Round the World

**Published:** 1916-01-08

**Authors:** 


### ROUND THE WORLD.

[The Editor Invites Early Information and Contributions Marked "Fellow-Workers."]

### Fellow-Workers and the Roll of Honour.

STAFF-SURGEON A. O. HOOPER, M.B., who lost his life in the explosion which took place on board H.M.S. *Natal*, entered the Service in 1912. He was the son of Dr. Alfred Hooper, of Gaddesby, near Leicester, and graduated in medicine at Edinburgh University in 1903. He held the post of assistant surgeon at Leicester Infirmary in 1903.

SURGEON D. W. K. MOODY, M.D., who was killed in the same explosion, was appointed temporary surgeon last August. He obtained his M.D. degree at Aberdeen University in 1902, having studied medicine in that city as well as in London, Dublin, and Berlin. He was formerly house surgeon at Peterborough Infirmary, and held resident appointments at the Central London Infirmary and Addenbrooke's Hospital, Cambridge.

CAPTAIN A. G. MILLER, R.A.M.C., attached 12th Middlesex Regiment, who is unofficially reported killed in France on December 29, was an Australian by birth. According to the *Times* he met his death during the course of his duties in the trenches, by a grenade discharged from a rifle.

LIEUTENANT A. B. THOMPSON, M.B., R.A.M.C., 3rd East Lancs Field Ambulance (T.F.), who is reported killed, obtained the L.R.C.P., L.R.C.S. (Edin.) in 1887, and subsequently practised medicine in the United States.

MAJOR F. M. JOHNSON, M.D., A.M.C., who, according to the *Times* of January 3, was killed at Anzac on November 29, was formerly in practice at Melbourne, Australia. He received his medical education at Melbourne, Edinburgh, Vienna, and Heidelberg, and obtained his M.D. at Edinburgh in 1888.

CAPTAIN C. A. BERNARD, R.A.M.C., attached 4th York and Lancaster Regiment (T.F.), who has been wounded in the North of France, was formerly in practice in Liverpool. He obtained his conjoint qualification L.R.C.P., L.R.C.S. (Edin.) in 1914.

SURGEON H. PARRY PRICE, R.N., contributes an interesting letter from Gallipoli to the current issue of *Guy's Hospital Gazette*. "Out here," he writes, "we wash in biscuit tins when we wash at all, and as to food, butter and any vegetable are almost unheard of luxuries. . . . Even in rest camps we live in small 'dug-outs,' and we are still under shell fire from the Turks, when they have any to spare."

DR. ARTHUR HILLYARD HOLMES, who went out to Serbia last October with the first British Red Cross unit organised by Sir Frederick Treves, has sent a message to his friends in Manchester stating that one hospital sister and himself are safe, and are travelling with Serbian officers to Durazzo. The telegram was sent from Elbassan, in Albania. All the rest of the hospital party, it is reported, are in the hands of the Austro-German troops.

SECOND-LIEUTENANT E. G. D. MILSOM, M.R.C.S., L.R.C.P., R.A.M.C., attached 7th Gloucestershire Regiment, is reported wounded. He is an old "Bart.'s" man, and at one time held resident appointments at the Belgrave Hospital for Children and the East Suffolk Hospital, Ipswich.

CAPTAIN H. W. WHITE, M.B., R.A.M.C., attached 11th Liverpool Regiment, who has been wounded, obtained his degree at Dublin in 1910. He has held various appointments, including that of assistant medical officer at Manor Asylum, Epsom, and house surgeon at the Metropolitan Hospital.

LIEUTENANT R. C. MCMILLAN, M.B., R.A.M.C., attached Welsh Regiment, 14th Battalion, has been wounded.

LIEUTENANT-COLONEL REGINALD PRATT, R.A.M.C. (T.F.), attached to the 5th Northern General Hospital, Leicester, the senior honorary physician to the Leicester Royal Infirmary, has been granted leave of absence from his infirmary duties for the period of the war. Colonel Pratt is the third honorary physician to the institution to seek leave of absence, Colonel Astley Clarke, A.D.M.S., and Major R. Sevestre having already been granted relief by reason of their military duties away from home. Major J. D. Slight, M.A., M.D. (Edin.), who has held several important medical appointments in large hospitals, and is a contributor to medical literature, has been appointed temporary physician to the institution in Colonel Pratt's absence.

